# Effect of Fenugreek Use on Fasting Blood Glucose, Glycosylated Hemoglobin, Body Mass Index, Waist Circumference, Blood Pressure and Quality of Life in Patients with Type 2 Diabetes Mellitus: A Randomized, Double-Blinded, Placebo-Controlled Clinical Trials

**DOI:** 10.31661/gmj.v8i0.1432

**Published:** 2019-03-30

**Authors:** Seyyedeh Seddigheh Hassani, Faramarz Fallahi Arezodar, Seyyed Saeid Esmaeili, Mohammad Gholami-Fesharaki

**Affiliations:** ^1^Department of Iranian Traditional Medicine, Faculty of Medicine, Shahed University, Tehran, Iran; ^2^School of Medicine, Immunoregulation Research Center, Shahed University, Tehran, Iran; ^3^Department of Biostatistics, Faculty of Medical Sciences, Tarbiat Modares University, Tehran, Iran

**Keywords:** Fenugreek Seed Meal, Diabetes Mellitus, Blood Glucose, Glycated Hemoglobin A

## Abstract

**Background:**

Diabetes mellitus is a major cause of death globally. It causes multiple problems in various organs and incurs heavy costs for patients and the community health system.The present study was conducted to evaluate the effect of fenugreek intake on fasting blood sugar (FBS), HbA1C, body mass index (BMI), waist circumference, blood pressure and quality of life of type 2 diabetes mellitus (T2DM) patients.

**Materials and Methods:**

This randomized, double-blinded clinical trial study was conducted on patients with T2DMin Tehran, Iran in 2018. The treatment group received 5 g of fenugreek powder,and the placebo group received 5 g of wheat flour twice daily for two months before meals.

**Results:**

This study was performed on 62 patients (50% male and 50% female). Both groups had similar demographic characteristics. The results showed a significant difference between the mean FBS (P<0.001), HgA1C (P<0.001), BMI (P<0.001), waist circumference (P<0.001), diastolic blood pressure (P=0.005), and quality of life (P=0.015). There was no significant difference in mean systolic blood pressure (P=0.189) between groups.

**Conclusion:**

Given the positive effect of fenugreek on FBS, hemoglobin, HbA1C, BMI, waist circumference, blood pressure and quality of life, it can be recommended for controlling blood glucose in diabetic patients.

## Introduction

Type 2 diabetes mellitus (T2DM) is a common metabolic disease [1]. It increased by 6.4% in 2010 to ~285 million and reached ~371 million in 2012. It has been estimated that the incidence of T2DM will increase to ~552 million by 2030 [2, 3]. Increased incidence of this disease is significantly correlated with an increase in age, change in lifestyle, overweight and economic status. The importance of T2DM is related to its high incidence and associated secondary problems which increase the cost of health care. T2DM is the leading cause of cardiovascular disease [4], end-stage renal disease [5], neuropathy and blindness in adults [6]. Strategies such as nutritional and physical activity, the use of glycemic and injectable drugs (insulin) have been recommended for the treatment of the disease. Oral administration of several drugs is associated with side effects such as diarrhea, nausea,bloating, hypoglycemia, overweight and hepatic damage [7]. Despite current treatments with drugs, blood glucose cannot be regulated to control the progression of the disease [8]. Studies have shown that 36% to 69% of patients fail to attain normal blood glucose, even after drug administration [9]. A better strategy without side effects is essential to control blood glucose in patients with T2DM. Recent investigations have considered the use of plant-based drugs as a natural, inexpensive and anti-diabetic agent without side effects for the treatment of diabetes [10-12]. Fenugreek, with the scientific name of *Trigonella foenum-graceum*, is an herbaceous annual that is native to the eastern Mediterranean. This is a common herbal plant for the treatment of diabetes. It is used frequently in different parts of the Mediterranean, especially in Iran [13]. In Iranian traditional medicine, fenugreek is considered to be blood glucose and fat reducing plant [14-16]. Studies have reported that fenugreek has therapeutic properties without side effects [17, 18]. Studies have shown that fenugreek is effective in reducing blood glucose and lipids [19-22]. It has been demonstrated that fenugreek reduces insulin tolerance by increasing the sensitivity of body cells to insulin [23]. Another study reports that the addition of fenugreek to bakery flour can control blood glucose [24]. Although many studies have considered the therapeutic effects of fenugreek on various diseases, less information is available about its effects on fasting blood sugar (FBS), HbA1C, body weight, waist circumference, blood pressure and quality of life in T2DM patients. In the current study, we considered the effect of fenugreek on these parameters in patients with T2DM.

## Materials and methods

### Sample Size Calculation

A total of 30 samples were calculated in each group based on $n=2{\left(Z_{\left(\frac{\alpha }{2}\right)}+Z_{\beta }\right)}^{2}/d^{2}$ where α=0.05, β=0.1 and *d*=0.6. If 10% of samples dropped, six samples were added to each group. Eventually, 36 samples were entered into each group.

### Drug and Intervention

Fenugreek, scientific name *T.foenum-graceum* (herbarium code from Shahid Beheshti Medical Science University: SMMU-8078) is a common anti-diabetic drug which can be cultivated in all seasons [11]. Fenugreek was purchased from a reliable market, ground, weighed using a balance (0.001 g accuracy) and packaged into opaque packets. A total of 120 packets (each containing 5 g fenugreek) were provided to each patient. Sixty packets were delivered to patients at the first meeting,and the other 60 packetswere delivered to them after one month of intervention. The placebo was providedsimilarly; however, wheat flour was used instead of fenugreek. The pharmacist provided the drug and placebo in the same packets, coded them and prepared the packets for the researcher. Only the pharmacist knew which packet contained the drug or placebo. The other researchers were blinded to it. Patients in the fenugreek group consumed 5 g of fenugreek powder and in the placebo group consumed 5 g of wheat flour twice daily for two months starting a meal.

### Patients

In this double-blinded, randomized clinical trial, the effect of fenugreek was investigated on the parameters of FBS, HbA1C, body weight, waist circumference, blood pressure and quality of life in T2DM patients. This study was conducted in Tehran, Iran from 22 November 2017 to 20 February 2018. T2DM patients referred to the Diabetes Association in Tehran, Health Center of Iranian Traditional Medicine (Shahed University) and private clinics were entered into the study. After the initial evaluation of 22,000, 5,000 and 8,000 medical documents at these locations, respectively, 1000 T2DM patients were selected according to the inclusion and exclusion criteria. After phone contact, only 260 patients agreed to participate in the study. Eventually, 144 of these patients were selected for further considerations based on the study criteria. Before the study, all selected patients visited medical centers in Tehran and consent letters were signed by all participants. The inclusion criteria werethe willingness to participate in the study, being 35 to 70 years of age, having at least a 6-month history of diabetes and body mass index (BMI)<35 kg/m2. Patients with use of glycemic drugs or insulin during the study, being pregnant orlactating, having an infectious disease (e.g., pneumonia, urinary infection, sepsis) with leukocytosis and neutrophilia, history of cancer, rheumatologic diseases, hormonal disease, drug addictions, smoking, being an organ transplant recipient were excluded from the study. At the time of examination, all patients were clinically stable and had not used any plant-based drugs. Patients who presented any complications such as unwillingness to participate, sensitivity to fenugreek, ketoacidosis, or chronic hyperglycemia were excluded from the study. A questionnaire containing demographic information was filled out by all patients,and the parameters of weight, height and waist circumference were measured. Blood pressure was measured by mercury barometer. FBS and HgA1C were measured using a glucose test apparatus (Easy Gluco, Infopia; South Korea) and Clover A1C analyzer (Infopia; South Korea), respectively. The quality of life was evaluated using a questionnaire comprising 36 questions. This self-report questionnaire included 8 subscales: physical function (PF), role impairment due to physical health (RP), role impairment due to emotional health (RE), energy/fatigue (EF), emotional well-being (EW), social function (SF), pain (P) and general health (GH). The questionnaire has been confirmed by numerous internal [25], and external [26] studies and a low score correlates with a poor quality of life.

### Groups and Study Design

The patients were randomly divided into two groups of treatment with fenugreek (n=36) and placebo (n=36) using the block randomization method (sequence AB, BA, by site; www.randomizer.org). Patients in both groups received standard treatments according to physician instructions. All patients received phone calls to obtain information about complications or improvements during the study. All patients in both groups were referred to our research center at the onset of the study and the second, third, sixth and eighth weeks to measure weight, FBS, HbA1C and waist circumference. In this study, the weight and waist circumference were measured at the study onset and on the sixth and eighth weeks and FBS and HgA1C were assessed at the onset and eighth week.

### Ethical Statesmen

The Ethics Review Board of Shahed University approved the study (IR.shahed.REC.1395.234). This study also registered and confirmed at Iranian Registry of Clinical Trials (RCT code: IRCT2017052233590N4).

### Statistical Analysis

In this study, the mean plus standard deviation (SD) of the descriptive statistics were used for continuous responses, and the frequency and percentage were used for categorical responses. For comparison between groups if the response was normal, the independent sample t-test was used and, if the response was not normal, the Man–Whitney U test was used. For a categoricalresponse, the Chi-square test was used for comparison between groups. For comparison within groups, the paired sample t-test and Wilcoxon test for normal and non-normal response were used, respectively. The data were normalized using the K-S test. Data were analyzed using SPSS software version 21(SPSS Inc., Chicago, IL, USA)and a P<0.05 was considered significant.

## Results

**Figure 1:**
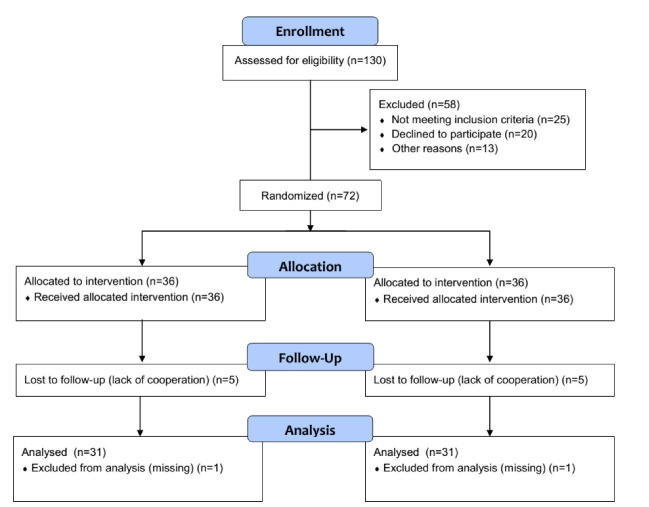
Flowchart of study

A total of 72 patients entered the study and 64 completed the survey (Figure-1). Table-1 shows the demographic data of all patients in each group. There was no significant difference for mean age, gender, educational level,and other demographic data between groups. The mean of FBS, HgA1C, BMI, waist circumference, systolic and diastolic blood pressure and quality of life scores before and after intervention in each group are shown in Table-2. The results indicate that the quality of life score in the treatment group increased significantly and the mean FBS, HgA1C, BMI, waist circumference, systolic and diastolic blood pressures decreased significantly. These changes, except for systolic blood pressure, were significant when compared with the placebo group.

## Discussion

Diabetes mellitus is a leading cause of mortality globally. It is associated with secondary problems in other tissues and incurs a heavy cost for patients and health care services [1]. An inexpensive and more effective strategy for the treatment of T2DM is required. We investigated the effect of fenugreek on FBS, HgA1C, BMI, waist circumference, systolic and diastolic blood pressure and quality of life in patients with T2DM. The data revealed that the quality of life score in the treatment group improved significantly after treatment with fenugreek. Interestingly, fenugreek significantly decreased the FBS, HgA1C, BMI, waist circumference, systolic and diastolic blood pressures in T2DM patients. These changes, except for systolic blood pressure, were significant when compared to those in the placebo group. The findings were compared with results obtained from previous studies [17, 24, 27, 28]. A clinical trial study by Kasaeian *et al*. [28] showed that fenugreek couldbe used as a medicinal plant along with other therapeutic methods for the treatment of T2DM. This therapeutic effect of fenugreek is related to the effects compounds such as steroids, alkaloids and trigonelline, which decrease Na-dependent absorption of glucose by the intestine [29, 30]. These compounds have antioxidant and anti-inflammatory properties which inhibit lipid peroxidation and other types of oxidation in an *in vitro* model [31, 32]. These chemical compounds inhibit free radical-induced tissue damage and prevent glycation, as a critical stage in cell proliferation, migration, disruption,and vascular endothelial cell death. Fenugreek can also be used to treat overweight and T2DM because of the anti-inflammatory properties of the compounds [33]. Our findings have also shown that fenugreek improves the quality of life of patients with T2DM, probably because of the management of the disease by fenugreek treatment. Studies have demonstrated that diabetes management can positively affect the quality of life of diabetic patients [34, 35]. The effects of anti-diabetic and cholesterol reduction by fenugreek seeds largely has been attributed to saponins and its high fiber content which delays gastric emptying and unknown components that constrain carbohydrate digestive enzymes [36]. Because fenugreek can be provided simply without adverse effects, this plant can be used for blood glucose control. The strength of the current study is related to its blinded status, evaluation of FBS, HgA1C, BMI, waist circumference, blood pressure and quality of life in T2DM patients with appropriate sample size. Its small sample size and the origin of the samples being only in one city are a limitation of this study.

## Conclusion

Fenugreek is effective for FBS and HgA1C control, lowering BMI, waist circumference, blood pressure and improving quality of life in T2DM patients. It can be ingested simply without adverse effects for blood glucose control in such patients.

## Conflicts of Interests

None.

**Table 1:** Some Clinical and Demographics Characteristics of Patients

**Categorical Variable** **N**		**Drug**		**Placebo**		**P-value**
		**%**	**N**	**%**		
**Gender**	Female	17	54.8	14	45.2	0.612
	Male	14	45.2	17	54.8	
**Education level**	Non-academic	28	90.3	21	67.7	0.059
	Academic	3	9.7	10	32.3	
**BMI**	≤25	7	22.6	13	41.9	0.174
	>25	24	77.4	18	58.1	
**Continuous variables**		**Mean**	**SD**	**Mean**	**SD**	**P-value**
**Age, y**		51.23	8.78	51.32	7.35	0.963
**Diabetes duration, y**		5.75	3.05	7.10	4.08	0.144
**WC (cm)**		98.05	9.83	95.05	8.56	0.305
**FBS (mg/dl)**		149.74	42.15	154.48	39.41	0.649
**HgA1C (mg/dl)**		7.96	1.80	7.86	1.51	0.830
**SBP (mm/Hg)**		86.81	11.34	80.94	10.35	0.795
**DBP (mm/Hg)**		133.68	15.24	137.94	13.15	0.346
		**Quality of Life score**		55.41	9.23	54.23	9.95	0.629

**SD:** Standard deviation, **WC:** Waist circumference, **SBP:** Systolic blood pressure, **DBP:** Diastolic blood pressure, **BMI:** Body mass index, **FBS:** Fasting blood glucose

**Table 2:** Comparison of Variables Before and After Intervention Between Groups

**Variables**	**Groups**	**Before**		**8 weeks later**		**Difference**	**P-value** ^*^	**P-value** ^**^
			**Mean**	**SD**	**Mean**	**SD**		
**FBS**	Drug	149.74	42.15	126.59	28.34	-23.15	<0.001	<0.001
	Placebo	154.48	39.41	153.58	37.11	-0.90	0.316	
**HgA1C**	Drug	7.96	1.80	7.46	1.35	-0.50	<0.001	<0.001
	Placebo	7.86	1.54	7.81	1.50	-0.05	0.033	
**BMI**	Drug	26.64	1.86	25.75	1.89	-0.89	<0.001	<0.001
	Placebo	25.31	1.91	25.21	1.87	-0.1	0.016	
**WC**	Drug	98.05	9.82	94.87	8.90	-3.18	<0.001	<0.001
	Placebo	95.05	8.56	95.00	8.65	-0.05	0.884	
**SBP**	Drug	133.68	15.24	127.77	10.60	-5.91	<0.001	0.189
	Placebo	127.94	13.15	124.84	13.57	-3.1	0.011	
**DBP**	Drug	86.81	11.34	81.03	7.17	-5.78	<0.001	0.005
	Placebo	80.94	10.35	50.48	9.25	-0.46	0.705	
**Quality of Life**	Drug	55.41	9.23	61.13	7.29	5.72	<0.001	0.015
	Placebo	54.22	9.25	55.46	9.7	1.24	0.197	

**SD:** Standard deviation, **WC:** Waist circumference, **SBP:** Systolic blood pressure, **DBP:** Diastolic blood pressure, **BMI:** Body mass index, **FBS:** Fasting blood glucose, *Before and After the intervention, **Between groups
